# Engineering of a calcium-ion binding site into the RC-LH1-PufX complex of *Rhodobacter sphaeroides* to enable ion-dependent spectral red-shifting

**DOI:** 10.1016/j.bbabio.2017.08.009

**Published:** 2017-11

**Authors:** David J.K. Swainsbury, Elizabeth C. Martin, Cvetelin Vasilev, Pamela S. Parkes-Loach, Paul A. Loach, C. Neil Hunter

**Affiliations:** a Department of Molecular Biology and Biotechnology, University of Sheffield, Firth Court, Western Bank, Sheffield, S10 2TN, United Kingdom; b Department of Molecular Biosciences, Northwestern University, Hogan 2100, 2205 Tech Drive, Evanston, IL 60208, United States

**Keywords:** BChl, Bacteriochlorophyll *a*, Crt, Carotenoid, CMac, CHCl_3_/CH_3_OH (1:1) containing 0.1 M ammonium acetate, CMC, Critical micelle concentration, EDTA, Ethylenediaminetetraacetic acid, HPLC, High performance liquid chromatography, LH1, Light harvesting complex 1, LH2, Light harvesting complex 2, *Rba.*, *Rhodobacter*, RC, Reaction centre, *Tch.*, *Thermochromatium*, UQ10, Ubiquinone 10, β-DDM, n-Dodecyl-β-d-Maltopyranoside, *Rhodobacter sphaeroides*, *Thermochromatium tepidum*, Protein engineering, Reaction centre, Antenna complex, Calcium-ion binding

## Abstract

The reaction centre-light harvesting 1 (RC-LH1) complex of *Thermochromatium* (*Tch.*) *tepidum* has a unique calcium-ion binding site that enhances thermal stability and red-shifts the absorption of LH1 from 880 nm to 915 nm in the presence of calcium-ions. The LH1 antenna of mesophilic species of phototrophic bacteria such as *Rhodobacter* (*Rba.*) *sphaeroides* does not possess such properties. We have engineered calcium-ion binding into the LH1 antenna of *Rba. sphaeroides* by progressively modifying the native LH1 polypeptides with sequences from *Tch. tepidum*. We show that acquisition of the C-terminal domains from LH1 α and β of *Tch. tepidum* is sufficient to activate calcium-ion binding and the extent of red-shifting increases with the proportion of *Tch. tepidum* sequence incorporated. However, full exchange of the LH1 polypeptides with those of *Tch. tepidum* results in misassembled core complexes. Isolated α and β polypeptides from our most successful mutant were reconstituted *in vitro* with BChl *a* to form an LH1-type complex, which was stabilised 3-fold by calcium-ions. Additionally, carotenoid specificity was changed from spheroidene found in *Rba. sphaeroides* to spirilloxanthin found in *Tch. tepidum*, with the latter enhancing *in vitro* formation of LH1. These data show that the C-terminal LH1 α/β domains of *Tch. tepidum* behave autonomously, and are able to transmit calcium-ion induced conformational changes to BChls bound to the rest of a foreign antenna complex. Thus, elements of foreign antenna complexes, such as calcium-ion binding and blue/red switching of absorption, can be ported into *Rhodobacter sphaeroides* using careful design processes.

## Highlights

• *Rba. sphaeroides* LH1 was engineered to include the Ca^2 +^ binding site from *Tch. tepidum.*
• Ca^2+^ binds with similar affinity to *Tch. tepidum* and engineered *Rba. sphaeroides* LH1.
• Ca^2+^ reversibly red-shifts a series of chimeric *tepidum*/*sphaeroides* LH1 subunits.
• LH1-RC assembly is required for reversible Ca^2 +^ shifting of LH1 absorption.
• Ca^2+^ enhances *in vitro* assembly of chimeric LH1 from α and β polypeptides and BChl.

## Graphical abstract

**Figure:**
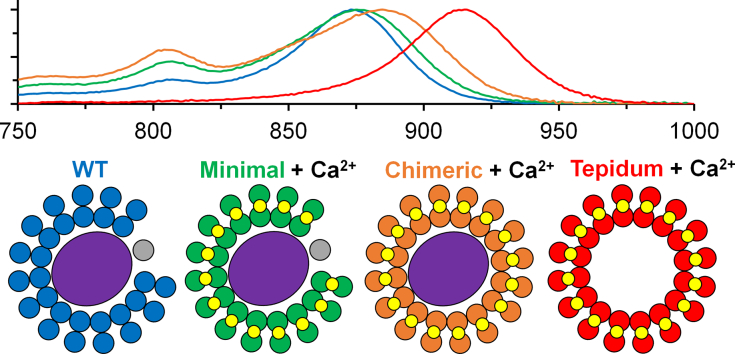

## 1 Introduction

The RC-LH1-PufX core complex of *Rhodobacter sphaeroides* (*Rba. sphaeroides*) is one of the most extensively characterised bacterial photosynthetic complexes. This is partly as a result of the long-established mutagenesis system in this organism [1], [2], which has allowed both the alteration of individual residues within the constituent proteins [3], [4], [5], [6], and the exchange of the carotenoids (Crt) for non-native alternatives [7], [8]. Using these altered complexes, the roles of key residues and interactions between the cofactors have been thoroughly characterised. Complementary to the mutagenesis studies are reconstitution studies [9], [10] where isolated pigments and proteins were modified and tested to examine their role in complex formation. From these experiments, necessary components were identified and the energetics of binding could be measured. In recent years the reaction centre (RC), RC-LH1-PufX and LH2 complexes have shown potential for integration into biohybrid devices for applications such as biosensing and biocomputing [11], [12], [13], [14], [15], [16], [17]. This organism may also have utility in the low-cost production of biofuels or high-value chemical products *via* genetic engineering, in which the RC-LH1-PufX core complex is central in providing energy during photosynthetic growth.

The core complex consists of the reaction centre (RC), which is formed of three polypeptides termed heavy (H), medium (M) and light (L). The RC binds a dimer of bacteriochlorophyll *a* (BChl *a*) molecules termed P870, two monomeric BChls, two pheophytins, two molecules of Ubiquinone10 (UQ10) and a single Crt. When light is absorbed by P870, or energy is transferred to it from other pigments, charge separation takes place leading to production of quinols and oxidized cytochrome *c* for downstream energy generation processes (for a comprehensive review of the RC see [18]). The RC is encircled by the light harvesting complex 1 (LH1) antenna, which captures light and channels resulting excitons to P870, increasing the absorbance cross-section of the RC. The LH1 of *Rba. sphaeroides* is comprised of 14 pairs of alpha (α) and beta (β) polypeptides, each of which forms a single transmembrane spanning alpha helix. The α polypeptides form an inner-ring in direct contact with the RC whilst β polypeptides form an external ring in contact with the lipid bilayer, or the peripheral light harvesting complex 2 (LH2) antenna. Between each αβ-pair, a dimer of BChls is bound with their central magnesium atoms coordinated *via* histidine residues [19], [20]. This packing arrangement along with other features of the binding sites, such as the dihedral angle of the hydrogen-bonded 3-acetyl group of BChl, causes a red-shift in absorbance from 760 nm when monomeric BChl in organic solvent to 875 nm in the LH1-bound state [21], [22]. Each αβ-pair also binds two Crt molecules, which broaden the wavelength range that can be harvested and provide protection against photo damage [23], [24].

The PufX component of the RC-LH1-PufX complex is comprised of a bent single transmembrane helix that prevents closure of the LH1 ring [20], [25], [26], [27]. This creates a channel allowing quinone/quinol to pass through the tightly-packed LH1 antenna and diffuse between the RC and the external quinone-pool [28]. It has recently been established that, in comparison with other core complexes with no PufX, the *Rba. sphaeroides* RC-LH1-PufX structure can bind more Crt molecules, enhancing the light harvesting and photoprotective ability of the LH1 antenna [29]. A second role of the *Rba. sphaeroides* PufX is to induce dimerisation of the core complex during photosynthetic growth. The two halves of the RC-LH1-PufX dimer are inclined towards each other [20], which is a major driver of membrane curvature leading to the formation of spherical intracytoplasmic membrane vesicles [30], [31].

Although light harvesting by the RC-LH1-PufX complex is already highly efficient, there are several major draw-backs when utilising it in man-made applications. One of these is the limited wavelength range that can be harvested as the pigments do not absorb in the red between 600 and 780 nm, nor beyond 900 nm. Another limitation is the stability of isolated complexes when utilised in bio-hybrid devices. Although devices based on RCs and RC-LH1-PufX complexes are delivering high current densities and good performance for herbicide biosensing, the lifetime of these devices remains relatively short, partly as a result of protein degradation (for a recent example see [16]).

Recent attention has been focused on overcoming these limitations through protein and genetic engineering. The light harvesting ability of the RC-LH1-PufX complex has been altered by introducing new carotenoid molecules into *Rba. sphaeroides* [32]. Another approach was the fusion of YFP to the RC-H subunit to enhance the light-harvesting ability of the RC-LH1-X complex [33]. Through protein engineering various pigments that extend the range of light absorption have been attached covalently or noncovalently to light-harvesting complexes and shown to efficiently transfer light energy [34], [35], [36], [37], [38], [39].

In this report we look to the diversity of RC-LH1 complexes of other organisms for inspiration to solve these shortcomings. Given the high sequence similarity of all LH1 polypeptides, we investigated whether desirable features could be utilised in a plug-and-play fashion to engineer new functionality into the *Rba. sphaeroides* complex.

The RC-LH1 core complex of thermophilic purple bacterium *Thermochromatium tepidum* (*Tch. tepidum*) is one example where useful functionality is present. This complex has a red-shifted LH1 antenna absorbing at 915 nm, distinct from the 850 nm absorbance of LH2 and extending the light-harvesting range of this organism much further into the infra-red than in *Rba. sphaeroides* [40], [41]. A second property of the *Tch. tepidum* complex is high thermal stability, which is an adaptation to allow photosynthetic growth at up to 55 °C in hot-sulphur springs [40], [42]. Both of these features are a result of the unique calcium ion-binding site in the C-terminal polar regions of the LH1 α and β polypeptides [43], [44]. The binding of calcium-ions stabilises the structure of the LH1 antenna, which in turn reduces thermal motion in the protein. The more rigid structure reduces the distance between BChl molecules, and the hydrogen bond to the BChl 3-acetyl group is shortened, which may also alter the dihedral angle of this moiety. The net-effect is inhomogeneous narrowing of the LH1 BChl absorbance band causing a further 35 nm red-shift and an increase in extinction [45].

In this work we engineer the LH1 polypeptides of *Rba. sphaeroides* to add a calcium-ion binding site using the *Tch. tepidum* sequence as a template. By producing a set of mutants in which incremental alterations were made with an increasing proportion of *Tch. tepidum* content, we have been able to gain insight into the requirements for the incorporation of calcium-ion binding. This study provides insights into the evolutionary requirements that give rise to the *Tch. tepidum* LH1 as well as the pitfalls and considerations required for transferring the functionality of foreign antenna systems into the LH1 antenna of *Rba. sphaeroides*.

## 2 Materials and methods

### 2.1 Sequence alignments

Amino acid sequences for *Rba. sphaeroides* PufB and PufA, and *Tch. tepidum* PufB and PufA were obtained from uniprotKB (accession numbers P0C0Y1, P0C0X9, D2Z0P1 and D2Z0P2, respectively). Sequences were aligned using NCBI Clustal Omega [46] and formatted using the ExPASy Boxshade server.

### 2.2 Gene design and strain generation

Genetic constructs were designed by reverse-translation of the desired peptide sequences and codon optimised for *Rba. sphaeroides* 2.4.1 using the Integrated DNA Technologies codon optimization tool. Synthetic sequences were used to replace the native *pufB* and *pufA* genes in a fragment of the *Rba. sphaeroides* genome spanning from 139 bp upstream of the start codon of *pufB*, and 18 bp downstream of the *pufA* stop codon *in silico* using A Plasmid Editor. This region encompasses upstream *Ahd*I and downstream *Fse*I restriction sites. The resulting DNA sequences were synthesised as GBlocks by IDT DNA. GBlocks were cut with restriction enzymes *Fse*I and *Ahd*I and the resulting fragments were ligated into a pre-existing construct of the suicide vector pK18mobsacBpufBAKO [47] (see supplementary Table 1 for plasmid sequence). The resulting plasmids contained the synthetic sequences flanked by 400 bp DNA sequences homologous to those directly upstream and downstream of the native *Rba. sphaeroides PufBA* genes. These were then conjugated into *Rba. sphaeroides* as described in [32], [48] using strain AH23 (Δ*pufBA*) or BC6 (Δ*pufBA*, Δ*puc1BA*, Δ*puc2BA*). The resulting strains contained the synthetic genes in place of the original pufBA genes in either a WT or LH2 minus background. The parent strains were generated by overlap extension PCR of the 400 bp upstream and downstream sequences of the target genes such that the coding sequences were absent from the final DNA fragment. These fragments were incorporated into pK18mobsac which was then used to replace the target genes in the *Rba. sphaeroides* genome. For a detailed description of the method see [32], [48]. Where multiple genes have been knocked out, gene deletion was performed sequentially until all target genes had been removed. An LH1 only strain was also generated by deletion of the *puc1BA*, *puc2BA* and *pufLMX* genes using the same method.

### 2.3 Semi-aerobic growth of cells

*Rba. sphaeroides* cells expressing the WT, Minimal, Chimeric and Tepidum LH1 in a background without LH2 were grown in 1.6 l flasks semi-aerobically in the dark in M22 + medium as described previously [49]. After 16 h of growth cells were harvested by centrifugation at ~ 4000 RCF for 30 min, drained of spent media and stored at − 20 °C until required.

### 2.4 Photosynthetic growth of cells

*Rba. sphaeroides* cells expressing the WT, Minimal, Chimeric and Tepidum LH1 in a background expressing LH2 were grown semi-aerobically in 80 ml M22+ media [49] for 24 h. Cells were used to inoculate three 17 ml screw-cap test tubes to give an OD at 680 nm of 0.03 for each strain in 16 ml of M22+ media. Tubes were illuminated at 30 μMol cm^− 2^ incandescent light from ORASM 116 W halogen bulbs. Cell cultures were continuously stirred to prevent settling of cells and OD readings were collected at 2, 6, or 12 h intervals over a period of 7 days.

### 2.5 Preparation of photosynthetic membranes

Cells were resuspended in 20 mM Tris pH 8 containing 5 mM CaCl_2_ a few crystals of DNAseI, Lysozyme and Roche cOmplete™ protease inhibitors. Cells were lysed by two passes through a French pressure cell (AminCo) at 20,000 PSI and insoluble material was removed by centrifugation at 18,459 RCF at 4 °C for 15 min. For spectroscopy membranes were prepared by loading supernatants onto 40/15% w/w sucrose step gradients and the membranes were separated by ultracentrifugation at 57,000 RCF at 4 °C for 10 h. Membrane bands were harvested from the interface of the two sucrose solutions, aliquoted and stored at − 20 °C until required. For protein purification supernatants were loaded directly into ultracentrifuge tubes and membranes were pelleted at 112,967 RCF at 4 °C for 3 h. Pelleted membranes were resuspended in 20 mM Tris pH 8 containing 5 mM CaCl_2_ by homogenisation and utilised immediately.

### 2.6 RC-LH1 core complex preparation

Homogenised membranes were solubilised in a solution of 20 mM Tris pH 8 containing 5 mM CaCl_2_ and 2% w/v n-Dodecyl-β-d-Maltopyranoside (β-DDM) in the dark at 4 °C with gentle mixing. Solubilised material was bound to a 50 ml DEAE Sepharose column (GE Healthcare) pre-equilibrated with 3 volumes of buffer A: 20 mM Tris pH 8 containing 5 mM CaCl_2_ and 0.03% w/v β-DDM. The column was washed with three column volumes of buffer A containing 130 mM NaCl (WT, Chimeric, Minimal) or containing 100 mM NaCl (Tepidum). Protein was then eluted over a linear gradient from 13 to 30% NaCl (WT, Chimeric, Minimal), or 10–20% NaCl (Tepidum). Peak LH1 containing fractions from each sample were pooled, diluted three fold and rerun on the column as described above. After two-rounds of ion-exchange fractions enriched in LH1 were pooled, concentrated to < 2 ml and further purified by size exclusion on a Superdex 200 16/30 GL column (GE healthcare) in buffer A containing 200 mM NaCl. LH1 containing fractions were pooled, concentrated, aliquoted and stored at − 20 °C until required.

### 2.7 UV/Vis spectroscopy

Spectra were collected in quartz semi-micro cuvettes in a Cary 50 or Cary 60 spectrophotometer over a wavelength range of 250–1000 nm (isolated complexes), or 350–1000 nm (membranes). The data interval was 0.5 nm and the averaging time was 0.1 s. Unless otherwise stated, spectra were collected in 20 mM Tris pH 8 containing 200 mM NaCl. Buffer for purified complexes also contained 0.03% w/v β-DDM. All buffers contained either 5 mM CaCl_2_ or 5 mM EDTA.

### 2.8 Fluorescence spectroscopy

Samples were prepared at an OD of 0.1 at the wavelength of the maximal Q_y_ absorbance in 20 mM Tris pH 8 containing 200 mM NaCl for membranes, or the same buffer with 0.03% w/v β-DDM for isolated complexes. Spectra were collected on a Fluorolog 2 (Horiba) fluorescence spectrophotometer equipped with a tungsten light source (ORSAM) and a Photocool series chilled PMT detector. Samples were excited at 590 nm with a slit width of 10 nm. Emission was recorded over a range of 800 to 1000 nm using an averaging time of 0.25 s, a slit width of 3 nm and a data interval of 0.5 nm. Spectra were the sum of 32 scans with corrections for detector sensitivity and lamp power applied. Data were processed by subtraction of the baseline, smoothed by performing a 5-point moving average and normalised using the absorbance of the same sample using Microsoft Excel.

### 2.9 Measurement of fluorescence lifetimes

Solutions of OD 0.5 at the wavelength of the maximal Q_y_ absorbance were prepared in either 20 mM Tris pH 8 containing 200 mM NaCl and 5 mM CaCl_2_ (membranes), or the same buffer containing 0.03% w/v β-DDM (isolated complexes). A volume of 10 μl of each sample was injected between a clean glass coverslip and a standard microscope slide separated by a 120 μm thick spacer.

Fluorescence lifetime measurements were performed on a home-built lifetime imaging microscope equipped with a spectrometer (Acton SP2558, Princeton Instruments), EMCCD camera (ProEM 512, Princeton Instruments) and a single photon hybrid photodetector (HPM-100-50 Becker & Hickl). A 485 nm pulsed laser (LDH-D-C-485, PicoQuant) with a repetition rate of 1 MHz was used as an excitation source. Fluorescence emission detection was filtered through a 605 nm dichroic mirror (FF605-Di02, Semrock) and either a 647 long-pass filter (BLP01-647R-25, Semrock), or a 900/32 nm band-pass emission filter (FF01-900/32-25, Semrock). A secondary slit in front of the single photon detector allowed further spectral narrowing of the measured signal; typically we were able to select ± 6 nm around the central wavelength selected by the monochromator and matched to the peak emission wavelength of the sample. The laser beam was focused on the sample surface to a diffraction limited spot using 100 × objective (PlanFluorite, NA = 1.4, oil immersion, Olympus), and the modulation of the laser was synchronised with a time-correlated single-photon counting (TCSPC) module (SPC-150, Becker & Hickl). Fluorescence lifetimes were recorded by parking the focused laser spot over a selected part of the sample surface and collecting data for 0.2–4 s; multiple measurements were performed on 10 to 12 different locations on each sample. SPCM software (Becker & Hickl) was used for the data acquisition. The families of decay curves were analysed with OriginPro and TRI2 software packages by fitting a multiexponential decay function:

$$I\left(t\right)=\sum_{i=1}^{n}A_{i}\exp\left(\frac{-t}{\tau_{i}}\right)+B$$

where *τ*_*i*_ is the fluorescence lifetime, *A*_*i*_ is the fractional amplitude contribution of the *i*th decay component, and *B* is the background. The quality of fit was judged on the basis of the reduced *χ*^2^ statistic:

$${\chi^{2}}_{\mathrm{red}}=\frac{\sum_{k=1}^{n}\frac{{\left[I\left(t_{k}\right)-I_{c}\left(t_{k}\right)\right]}^{2}}{I\left(t_{k}\right)}}{n-p}=\frac{\chi^{2}}{n-p}$$

where *I(t*_*k*_) is the data at time point *k*, *I*_*c*_(*t*_*k*_) is the fit at time point *k*, *n* is the number of data points and *p* is the number of variable fit parameters (*n*-*p* = degrees of freedom).

The instrument response (IRF) of the system, measured using a mirror instead of a sample, was approximately 130 ps, and the convolution of the decay curves with the IRF was taken into account when the fitting was performed.

### 2.10 Purification of α and β polypeptides

Chromatophores were prepared from whole cells by adaptation of the procedure of Parkes-Loach et al. [50]. Whole cells were pelleted by centrifugation at 4000 RCF for 20 min and washed once with deionized water. They were then resuspended in 30 ml of 50 mM Tris buffer, pH 7.5, and sonicated in an ice bath for 14 min at a power setting of 7 using a Branson Sonifier Cell Disrupter 200. Unbroken cells and cellular debris were pelleted by centrifugation at 27,200 × g for 30 min. The membrane fraction was collected by spinning the supernatant at 111,000 × g for 60 min, resuspending the pellet in Tris buffer and recentrifuging at 111,000 × g for 60 min, and finally washing with deionized water and centrifuging at 111,000 × g for 60 min. The membranes were lyophilized to dryness and stored at − 20 °C until required.

Lyophilized membranes (100–200 mg) were extracted with 4 ml of CHCl_3_/CH_3_OH (1:1) containing 0.1 M ammonium acetate (CMAc). After a 5 min centrifugation in a tabletop centrifuge, the supernatant was carefully removed and the pellet was subjected to a second extraction with an additional 2 ml of CMAc. After centrifugation for 5 min, the supernatant was added to that of the first extraction, and the combined extract was applied to a Sephadex LH60 gel filtration column (50 cm × 2.5 cm) using CMAc as the eluting solvent for separation of the RC, LH1 α and β polypeptides, pigments and lipids. The separation of components is indicated in supplementary Fig. 3 a–c for the *Rba. sphaeroides puf*BA extracts from the *Rba. sphaeroides* puc705-BA (WT LH1 + LH2 −), Chimeric and Tepidum strains. The region containing the α and β polypeptides was divided into three fractions: early, middle and late. Each pooled fraction was immediately evaporated to dryness under a stream of argon, further dried overnight on a lyophilizer, and then stored at − 20 °C. A portion of each sample was dissolved in 10–30 μL of hexafluoroacetone trihydrate (HFA) with sonication, an equivalent volume of HPLC 1:1 A/B solvent was added, the sample was centrifuged, and the supernatant injected into the HPLC. Solvent A was composed of deionized water containing 0.1% trifluoroacetic acid and solvent B was 2:1 acetonitrile:isopropanol containing 0.1% trifluoroacetic acid. A Perkin-Elmer HCODS C18 column was used and the gradient started at 50% B and moved linearly to 70% B at 10 min, 90% B at 30 min, 100% B at 32 min, staying at 100% B until 34 min then back to 50% B at 36 min and remained at 50% B for 10 min. The separation of peaks containing the α and β polypeptides are shown in supplementary Fig. 3 d–f for the Chimeric and Tepidum mutants. Profiles for the Minimal mutant were similar to that for *Rba. sphaeroides* (data not shown). The appropriate peaks were collected, the solvent evaporated under argon and the samples further dried on the lyophilizer overnight.

### 2.11 *In vitro* reconstitution of B820 and LH1-type complexes

The conditions for forming αβ-subunits (B820-type) and oligomers (LH1-type) have been previously described [34], [35] and were followed with a few changes. The buffer used for these experiments was changed to 0.05 M Tris, pH 8.5 because phosphate-containing buffer would precipitate when Ca^2 +^ is added. In addition, the reconstitution is very sensitive to ionic strength so that the Tris buffer, pH 8.5 was prepared by adding solid in the amount 435 mg Tris (the base) and 221 mg Tris hydrochloride (the acid) to minimize the amount of chloride required. Control reconstitution experiments using the LH1 polypeptides from *Rba. sphaeroides puc*705BA showed no difference in B820 and LH1 formation whether the original potassium phosphate buffer or the Tris buffer was used. Once BChl was added, the detergent solution sample was diluted to optimize formation of the αβ-subunit (B820) and then, if adding Ca^2 +^, an amount of 1.0 M CaCl_2_ was added until the concentration was 5 mM. LH1-type oligomers were formed by incubation of the samples at 4 °C.

### 2.12 Generation of calcium-ion free complexes

Calcium-ions were removed to generate ion-depleted proteins based on a method by Swainsbury et al. [51]. Solutions of 1 M HCl, 1 M Tris pH 8, 5 mM Tris pH 8 and 5 mM Tris pH 8 containing 0.03% w/v β-DDM were prepared in plasticware extensively washed in 1 M HCl followed by MilliQ water. A 5 ml chelating Sepharose column (GE healthcare) was washed with two volumes of 1 M HCl to remove bound ions. The pH was restored by washing with 1 volume 1 M Tris pH 8 then equilibrated with two volumes 5 mM Tris pH 8 (membranes) or 5 mM Tris pH 8 containing 0.03% β-DDM (Pure proteins). Membranes or pure protein were concentrated to < 0.25 ml if required, loaded onto the column and eluted in 10 ml of the equilibration buffer.

### 2.13 Reconstitution with calcium-, strontium- and barium-ions

Calcium-ion depleted proteins were incubated in 5 mM CaCl_2_, SrCl_2_ or BaCl_2_ at room temperature in the dark for 30 mins. Spectra were collected between 1000 and 250 nm.

### 2.14 Measurement of calcium-ion binding affinity

Calcium-ion depleted proteins were diluted to a maximum OD of 0.1 at the wavelength of the maximal Q_y_ absorbance and spectra were collected between 650 and 1000 nm. Injections of a 250 μM solution of CaCl_2_.2 H_2_O (prepared from a fresh, factory sealed powder of > 99.5% purity, Sigma) were performed to give a range of final concentrations of 0.16–48 μM and spectra were collected after each. Data were processed by plotting the absorbance ratio of points at 885 and 875 nm and fitted to a single binding site model in graph pad prism. Three datasets were collected for each sample.

### 2.15 Thermal denaturation

Isolated complexes were added to solutions of 20 mM Tris pH 8 containing 0.03% w/v β-DDM and either 5 mM CaCl_2_ or 5 mM EDTA pre-heated at 55 °C. Denaturation was monitored by the loss of the distinctive LH1 absorbance band over a 90 minute period by collecting spectra between 650 and 1000 nm with a 1 nm interval and 0.0125 s integration time. Three data sets were collected for each sample in each buffer.

## 3 Results and discussion

### 3.1 Design of calcium-ion binding RC-LH1 complexes

Using the crystal structure and amino-acid sequences of the *Tch. tepidum* RC-LH1 complex as a guide, we were able to identify key features of the α and β polypeptides from *Tch. tepidum* that differ in the homologues of *Rba. sphaeroides*. In *Tch. tepidum* LH1, the calcium-ion binding site is formed by the C-terminus of the β polypeptides and a polar loop of amino acids in the α polypeptides [44]. Ligands are provided by the two C-terminal oxygens of the β-polypeptide, the backbone oxygen of αTrp + 10, and sidechain oxygens of αAsp + 13 and αAsn + 14 (numbering is relative to the chelating histidine residues of each polypeptide) [44]. The 3.0 Å structure is of insufficient resolution to resolve whether additional ligands may be provided by two water molecules.

**Fig. 1:**
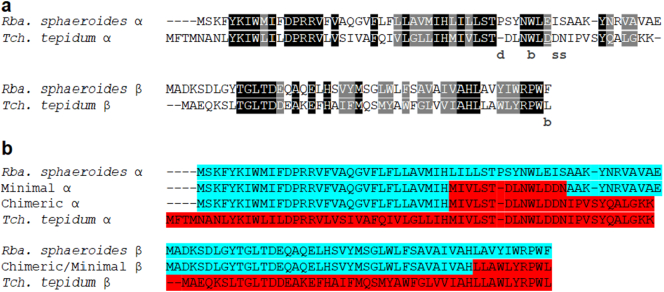
a. Sequence alignments of the *Rba. sphaeroides* and *Tch. tepidum* LH1 α and β polypeptides. Black highlights show conserved residues and grey shows similar residues. Annotations below the sequence denote deletions (d), and calcium-ion chelating residues *via* backbone oxygens (b) or side-chains (s). b. Alignments of α and β peptides from *Rba. sphaeroides* and *Tch. tepidum* (coloured blue and red, respectively), and the designed Chimeric and Minimal peptides, retaining the highlighting of the parent sequences.

Fig. 1 a shows sequence alignments of the *Rba. sphaeroides* and *Tch. tepidum* α and β polypeptides. As there is no mutagenesis system in *Tch. tepidum*, the residues required for calcium-ion binding and red-shifting of the antenna absorbance have not been biochemically confirmed, so we used the alignments and structural analysis to design our hybrid peptides. Examination of the alignments reveals that a deletion is present in the *Tch. tepidum* α polypeptide, which aligns with the αPro + 7 on of *Rba. sphaeroides*. This deletion may be essential for allowing a viable calcium-ion binding conformation of the loop. We also observe that the side-chain chelating αAsp + 13, and αAsn + 14 are substituted for Ile and Ser in the *Rba. sphaeroides*. The C-terminal domains of the LH1 β polypeptides also differ and might allow this region to adopt an appropriate conformation for chelation. As calcium-ion binding has a notable effect on the properties of the bound BChls, we hypothesised that the region between the Mg-chelating histidines and calcium-ion binding residues could also be important. With these considerations in mind we designed a minimal construct in which the residues from the chelating histidine to the end of the binding regions were altered to match the *Tch. tepidum* sequence. In addition we produced a chimeric construct where the entire C-terminal sections were altered to match *Tch. tepidum*. Finally, we produced a complete exchange of the *Rba. sphaeroides* LH1 polypeptides for those of *Tch. tepidum*. Amino acid sequences of the designed peptides are shown in Fig. 1b.

### 3.2 Expression and purification of engineered RC-LH1 complexes

The designed DNA sequences were integrated into the genome of a *Rba. sphaeroides* strain in which the LH2 encoding *puc1BAC* and *puc2BA* genes have been deleted (see Materials and methods section); this allows for simple spectroscopic characterisation of the complexes as the overlapping absorbance of LH2 is eliminated. The resultant strains are named WT for the wild-type *Rba. sphaeroides* LH1, Minimal for the strain containing Minimal LH1, Chimeric for the chimeric LH1 and Tepidum for the strain with *Tch. tepidum* LH1 expressed in *Rba. sphaeroides* (Fig. 1b).

**Fig. 2:**
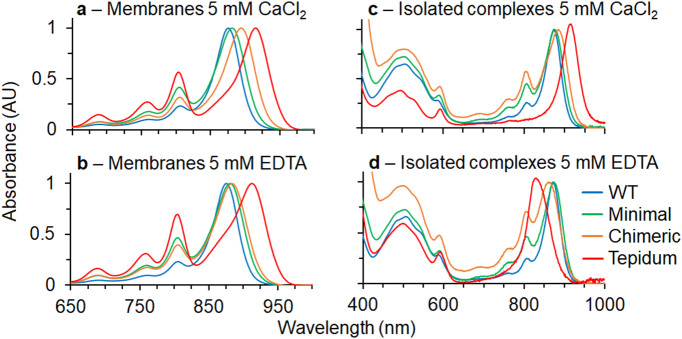
Spectra of *Rba. sphaeroides* LH1 complexes from an LH2-deficient strain expressing WT (blue), Minimal (green), Chimeric (orange) and Tepidum (red) LH1 polypeptides. Spectra are shown for membranes in the presence of a: 5 mM CaCl_2_ and b: 5 mM EDTA, and purified complexes in the presence of c: 5 mM CaCl_2_ and d: 5 mM EDTA.

After 16 h of growth under dark, semi-aerobic growth conditions cell absorption spectra showed evidence of LH1 complexes with a maximum red-shifted relative to WT. We note that the expression level of the LH1 is reduced between two- and five-fold compared to that of the wild-type for all mutants based on LH1 absorbance in whole-cell spectra (data not shown). This is similar to an observation by Fulcher and co-workers when expressing the *Rba. capsulatus* LH1 in *Rba. sphaeroides* [52]. To produce higher quality spectra and generate material suitable for further characterisation, LH1 containing membranes were isolated. Spectra of these membranes after preparation in excess CaCl_2_ reveal that the LH1 absorbance is red-shifted by 6, 19 and 41 nm relative to WT for the Minimal, Chimeric and Tepidum constructs, respectively (Fig. 2a and Table 1). When the isolated complexes are incubated in excess ethylenediaminetetraacetic acid (EDTA) to remove all bound calcium-ions, we observe blueshifting of the Minimal and Chimeric LH1s by 1 and 11 nm, respectively (Fig. 2b and Table 1). This shift moves the absorbance maximum close to the position of the WT. A similar absorbance maximum has previously been reported for the *Tch. tepidum* RC-LH1 complex in EDTA [43]. As expected, no spectral differences were observed for WT in excess CaCl_2_ or EDTA. Surprisingly, the Tepidum LH1 in membranes displayed a limited blue-shift of 3 nm to 913 nm. Reasons for this apparent insensitivity to calcium-ion removal will be discussed in later sections. These engineered LH1 polypeptides show that a minimal set of alterations can impart calcium-ion binding to LH1 complexes that lack this ability. However, properties of the complete *Tch. tepidum* α and β polypeptides are required for the full red-shift to 915 nm. This suggests that features of the N-terminal regions of the α and β polypeptides that were not incorporated into the Minimal and Chimeric constructs also play a role in the calcium-ion dependent red-shift of *Tch. tepidum* LH1.

**Table 1:** Absorbance wavelengths of Q_y_ bands for LH1 in membranes and for purified complexes.

Sample	Preparation	Q_y_ CaCl_2_	Q_y_ EDTA
WT	Membranes	875	875
Minimal		881	880
Chimeric		894	883
Tepidum		916	913
WT	Pure complexes	873	873
Minimal		875	875
Chimeric		885	863
Tepidum		915	831

To gain further insight into the properties of our constructs, we solubilised membranes using n-Dodecyl-β-d-Maltopyranoside (β-DDM) and purified RC-LH1 complexes by ion-exchange and size-exclusion chromatography. Spectra of the isolated complexes are shown in Fig. 2c and d and peak wavelengths are summarised in Table 1. During the preparation, a large quantity of free RCs were observed suggesting that assembly of our RC-LH1 complexes is somewhat impaired. Nevertheless for the Minimal and Chimeric constructs we were able to enrich RC-LH1 core complexes, albeit with an attenuated LH1 absorbance. Interestingly the *Tch. tepidum* LH1 did not co-purify with RCs suggesting that the LH1 of *Tch. tepidum* is incompatible with the RC of *Rba. sphaeroides*. Further experiments (discussed below) provide evidence that this is also the case in the native membranes and not an artefact of detergent treatment. The absorption maxima of purified Minimal and Chimeric LH1 complexes are similar to those of native membranes. In excess CaCl_2_ the Q_y_ band of these LH1 complexes is red-shifted by 2 and 12 nm from the WT, respectively. After incubation in excess EDTA the absorbance blue-shifts to a similar position to the WT. Different behaviour was observed for the Tepidum complex, which absorbs at 913 nm in CaCl_2_. However, in EDTA this peak blue-shifts to 830 nm, similar to the B820 assembly intermediates observed when LH1 complexes are disassembled into LH1 αβBChl_2_ subunits [9]. This is in contrast to the Tepidum membranes, which were insensitive to EDTA treatment. These data suggest that the free Tepidum LH1 antenna is unstable in the calcium-ion-free state when detergent solubilised, perhaps not surprising given that the RC and antenna of any given organism will co-evolve giving rise to specific interactions that stabilise the complex.

**Fig. 3:**
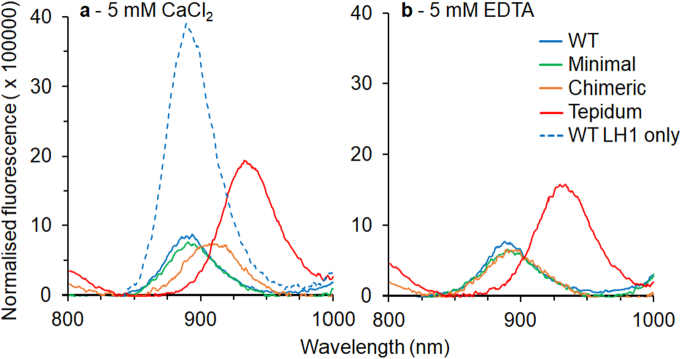
Fluorescence spectra of *Rba. sphaeroides* membranes from an LH2-deficient strain expressing WT (blue, solid line), Minimal (green), Chimeric (orange) and Tepidum (red) LH1 polypeptides. An additional experiment of WT LH1 in a strain also lacking reaction centres is shown in the presence of CaCl_2_ (blue dashed line). Spectra are shown for membranes in the presence of a: 5 mM CaCl_2_ and b: 5 mM EDTA. Excitation was at 590 nm. Spectra shown are the sum of 32 traces.

To probe energy transfer from the LH1 complexes to the RC we recorded fluorescence spectra and measured fluorescence lifetimes. As shown in Fig. 3, the emission spectra are red-shifted in membranes for the Minimal and Chimeric constructs. In EDTA the spectra blue-shift towards the WT position, as was observed for the absorbance spectra. Similar results were obtained for the isolated complexes (data not shown). As with absorbance of the membranes, the Tepidum construct appears to be insensitive to calcium-ion removal. Another feature of the Tepidum spectrum is the increased fluorescence yield (approximately 2-fold) relative to WT, Minimal and Chimeric (note all samples had an identical max Q_y_ absorbance of 0.1). An increased yield is also observed for membranes prepared from a strain in which the RC has been removed from the WT LH1 (WT LH1-only), which prevents energy transfer to the RC. The reason for the difference in fluorescence yield between the WT LH1-only control and Tepidum construct could not be determined. We suspect that there may be a reduction of the fluorescence caused by the red-shift or instability of the construct. It is also possible that a low level of energy transfer may persist leading to a quench of fluorescence.

To further analyse the energy transfer and overcome potential difficulties in interpretation of the steady-state fluorescence measurements, we measured the fluorescence lifetimes of the mutants as summarised in Table 2 (see supplementary Fig. 1 for raw traces). When purified and in membranes, the Minimal and Chimeric complexes display similar lifetimes to the wild-type of ~ 300–400 ps. This suggests that energy transfer to the RC is not impaired in these constructs. Purified Tepidum, which is free of RCs, displays a much longer lifetime of 842 ps, which suggests that the extended lifetime of this complex arises from loss of energy transfer to the RC. The same result is seen in membranes, suggesting that energy transfer to the RC is also impaired *in vivo*. As an additional control we measured the fluorescence lifetime of the WT LH1-only membranes and observed a similar lifetime to the Tepidum construct. Together these data demonstrate that the Minimal and Chimeric constructs form functional core complexes *in vivo*, whereas the Tepidum LH1 is unable to assemble in a suitable fashion to permit energy transfer to the *Rba. sphaeroides* RC. This is in contrast to the expression of *Rba. capsulatus* LH1 in *Rba. sphaeroides* where complete RC-LH1 core complexes were assembled [52], [53]. This is likely to be a result of the *Tch. tepidum* LH1 being much more distantly related than that of *Rba. capsulatus* and thus lacking residues responsible for key interactions with the RC to stabilise the core complex, which are preserved in the *Rhodobacter* species.

**Table 2:** Fluorescence lifetimes of complexes in native membranes and purified in β-DDM.

Sample	*A*1	*τ*1 ± sd [ps]	*A*2	*τ*2 ± sd [ps]	*χ*_red_^2^
WT pure	5673	295 ± 40	407	853 ± 139	1.05 ± 0.15
WT membranes	10,380	264 ± 7			1.05 ± 0.11
Minimal pure	5100	331 ± 13			1.04 ± 0.16
Minimal membranes	3633	367 ± 15			1.10 ± 0.13
Chimeric pure	4092	314 ± 8			0.99 ± 0.14
Chimeric membranes	5246	313 ± 10			1.08 ± 0.17
Tepidum pure	1621	842 ± 20			1.01 ± 0.19
Tepidum membranes	1855	713 ± 18			0.92 ± 0.12
WT LH1-only membranes	2251	765 ± 13			1.01 ± 0.14

As all of our designed strains contain the *pufX* gene, we used immunodetection to see if PufX was incorporated into the complexes. Supplementary Fig. 2 shows western blots of membranes and pure complexes probed with an anti-PufX antibody [54]. The data show that PufX is present in all strains. PufX was also detected in purified WT and Minimal complexes; however, PufX was not detected in the purified Chimeric and Tepidum complexes. This can be rationalised by the previous finding that PufX interacts with the C-terminal region of the *Rba. sphaeroides* α subunit [27], [55], [56]. As the Chimeric and Tepidum constructs both have the entire C-terminal region of α exchanged for a sequence from a species that does not have a PufX-like gene, it is likely that key residues mediating this interaction have been removed or changed.

Based on the data discussed in this section, we expect that the WT and Minimal LH1 antenna surround the RC, and the PufX polypeptide is present. We expect that the Chimeric LH1 forms an incomplete ring surrounding the RC (see graphical abstract for a schematic representation). Inspection of Fig. 2A shows that the LH1:RC (875:800 nm) absorption ratio is only half that of the WT complex, probably as a combined result of the free RCs identified during ion-exchange and attenuation of the LH1 absorbance observed in pure complexes. In both membranes and when isolated in detergent it is clear that the Tepidum LH1 assembles without the RC. This LH1-only complex is unable to undergo the conformational changes required for blue shifting to ~ 880 nm upon calcium-ion removal. These findings suggest that exchanging the native *Rba. sphaeroides* LH1 for the *Tch. tepidum* antenna complex in a plug and play fashion leads to some difficulty in assembly with the *Rba. sphaeroides* RC. By utilising a more incremental design process, such as that employed for the Minimal and Chimeric constructs, we have overcome some of these assembly issues to impart new functionally to the existing antenna.

### 3.3 *In vitro* assembly of complexes

To gain further insight into the assembly of the Minimal, Chimeric and Tepidum LH1 complexes, the α and β polypeptides from each strain were purified, and complex formation was monitored following *in vitro* reconstitution with BChls [9], [10]. The separation of peptides from the Chimeric and Tepidum constructs after solvent extraction are compared in supplementary Fig. 3 a–c. Data for the Minimal peptide purification are not shown but had a similar profile to that of the WT-LH1 containing strain with a yield between that of WT and Chimeric. The lower yields of polypeptides isolated from the Minimal, Chimeric and Tepidum constructs reflect the lesser expression in these three strains compared to the WT. As can be seen from the high performance liquid chromatography (HPLC) separation of the chimeric polypeptides in supplementary Fig. 3 d and e, the α polypeptide elutes before the β polypeptide, as is the case for WT and Minimal polypeptides with similar retention times to the WT. The results of the extract from Tepidum show an inversion of the locations of the α and β HPLC peaks with the β polypeptide eluting first and two prominent α polypeptide peaks occurring second (supplementary Fig. 3 f). The same pattern was reported by Wang et al. [57] using a similar procedure.

**Fig. 4:**
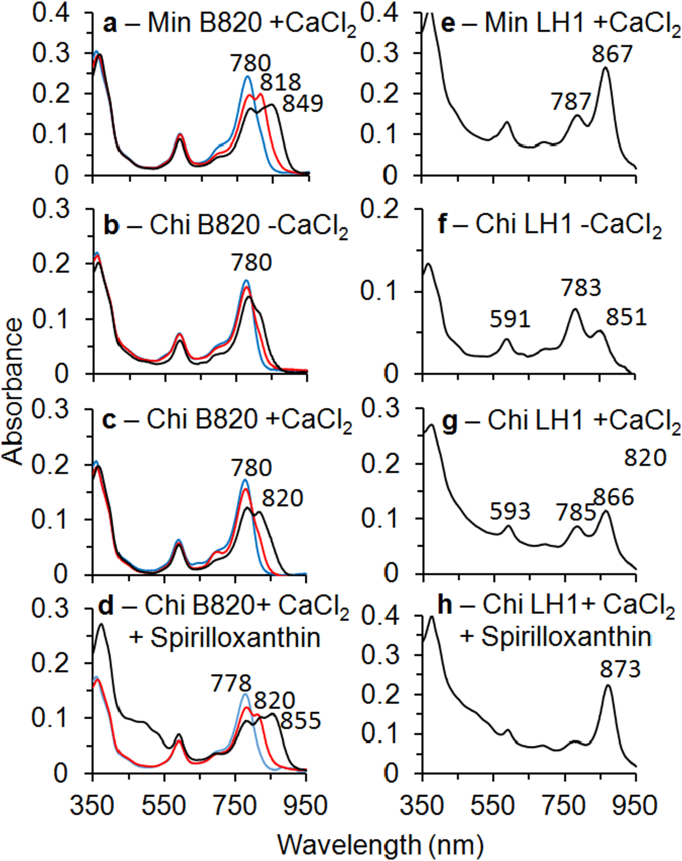
a–d: Formation of B820 complexes in the presence of 0.9% (blue), 0.75% (red) and 0.64% (black) β-OG detergent for α and β polypeptides from a: Minimal in CaCl_2_. b: Chimeric in the absence of CaCl_2_, c: Chimeric with CaCl_2_. d: Chimeric with CaCl_2_ and spirilloxanthin. e–h: Formation of LH1-type complexes at low ionic strength for α and β polypeptides from e: Minimal in CaCl_2_. f: Chimeric in the absence of CaCl_2_, g: Chimeric in CaCl_2_ and H: Chimeric in CaCl_2_ with spirilloxanthin. Peaks are labelled with their respective absorbance wavelengths.

Using the α and β polypeptides isolated from the Minimal strain with 5 mM CaCl_2_ present, B820- and LH1-type complexes were formed as shown in Fig. 4a and e, although not as completely as with the WT polypeptides from *Rba. sphaeroides* [58]. It should be noted that unlike the pattern followed by reconstitution of *Rba sphaeroides* LH1, the subunit complex partially formed at 0.75% OG is red-shifted at 0.64% OG to 849 nm. This is presumably due to calcium binding displacing the equilibrium towards LH1. When the reconstitution with the Minimal polypeptides is done in the absence of calcium (data not shown), the B820 subunit remains at 818 nm until the sample is chilled under LH1-forming conditions. Thus, calcium binding has an effect on the reconstitution by promoting LH1 formation but does not enhance the amount of LH1 formed. The Q_y_ maximum for the LH1-type complex was at 867 nm, characteristic of the *Rba. sphaeroides* reconstituted WT LH1.

Using polypeptides from the Chimeric construct a B820-type complex did not form well in the absence of calcium nor did an LH1-type complex (Fig. 4b and f). In the presence of 5 mM CaCl_2_, a B820-type complex was more distinctly formed as was an LH1-type complex (Fig. 4c and g). This reconstitution was very sensitive to ionic strength which was kept as low as was consistent with maintaining a buffered pH (0.02 M). When repeated at a higher ionic strength (about 3-fold), no B820 or LH1 formed in the absence of Ca^2 +^ and only about half as much of each formed as in Fig. 4f in the presence of Ca^2 +^. Note that the Q_y_ maximum for the LH1-type complex formed in Fig. 4g was 866 nm. Thus, calcium-ion dependent formation of B820 and LH1 was observed (enhanced about 3-fold relative to without Ca^2 +^) but there was no additional red-shift of the Q_y_ maximum compared to WT *Rba. sphaeroides* LH1. There are likely some additional interactions necessary to red-shift the absorption spectrum that are absent in the *in vitro* system. One possibility is that the carotenoid must be included in the LH1 complex. Reconstitution experiments with the Chimeric polypeptides and the carotenoid spheroidene either failed to enhance LH1 formation or had only a minimal effect (data not shown). This is a substantial change from reconstitution with WT *Rba. sphaeroides* polypeptides in which the B820 complex is strongly shifted to the LH1 oligomer even at room temperature and above the critical micelle concentration (CMC) of OG [59].

However, the native carotenoid in *Tch. tepidum* is spirilloxanthin, which may exhibit different effects on binding. Reconstitution experiments with the Chimeric polypeptides with 5 mM calcium and the carotenoid spirilloxanthin gave a very different result (Fig. 4d and h). At standard B820 forming conditions the sample was substantially red-shifted to 855 nm even at 0.75 and 0.64% OG at room temperature and LH1 was more quantitatively formed below the CMC than in the absence of spirilloxanthin (Fig. 4g) and the Q_y_ was red-shifted about 7 nm. Although a significant red-shift of the BChl Q_y_ band and a pronounced stabilisation of reconstituted LH1 has resulted from spirilloxanthin binding, the Qy maximum is still at a much shorter wavelength than that of *Tch. tepidum* LH1. To induce red shifting of the chimeric LH1 to 885 nm, additional components, such as bound lipids, may also be required. An additional possibility that cannot be ruled out is that the calcium-ion binding in *in vitro* reconstituted complexes may not be identical to the *in vivo* assembled proteins. There are two crystal structures of the Tepidum LH1-RC core complex that are relevant here. The first was prepared in the presence of excess Ca^2 +^ [44] and the second was prepared with Sr^2 +^ or Ba^2 +^ substituted for Ca^2 +^ [60]. In the first, the ligands coordinated to Ca^2 +^ contain two oxygen atoms from the C-terminal α carboxylate of the β polypeptide and the rest are from amino acids of the α polypeptide and possibly water, whereas all amino acids providing ligands in the second structure are from α polypeptides and possibly water. The Q_y_ band in the second is at 888 nm instead of 915 nm and the complex is less stable due to a lack of the inter subunit coordinating ligands to Ca^2 +^. Thus, the Ca^2 +^ binding motif in LH1 reconstituted with the α and β polypeptides of the Chimeric mutant may have a structure similar to that of the Sr^2 +^ prepared complexes that lack a significant red-shift of the BChl Q_y_ band.

*The change* in the *Rba. sphaeroides* β polypeptide amino acid sequence for the Minimal and Chimeric mutants was minimal because there are only 10 amino acids from His 0 to the C-terminus, four of which are identical to those in the *Tch. tepidum* sequence and three of the other six are conservative changes (Fig. 1b). Therefore, it is not surprising that the isolated β polypeptides of the Minimal and Chimeric mutants would associate in the absence of the α polypeptide to form a ββ homodimer although less stably so than the *Rba. sphaeroides* WT ββ homodimer (data not shown). Use of the *Rba. sphaeroides* β polypeptide together with either the minimal or chimeric α polypeptide did not form a stable B820-type complex and failed to form a LH1-type complex. Thus, one or more of the six altered amino acids make a significant difference to forming a LH1-type complex when paired with the Minimal or Chimeric α polypeptide. This suggests that the α and β polypeptides of LH1 have co-evolved to maintain their interactions and these must be preserved during the design of engineered hybrid complexes. Reconstitution experiments with the polypeptides from the Tepidum construct have been initiated but suitable conditions for their reconstitution have not yet been identified.

### 3.4 Ion-binding properties of isolated complexes

To gain further insight into the ion-binding properties of our engineered complexes, and allow a more thorough comparison with the template *Tch. tepidum* RC-LH1 complex, we examined the binding of alternative ions, the calcium-ion binding affinity, and the influence of calcium-ions on thermal stability.

**Fig. 5:**
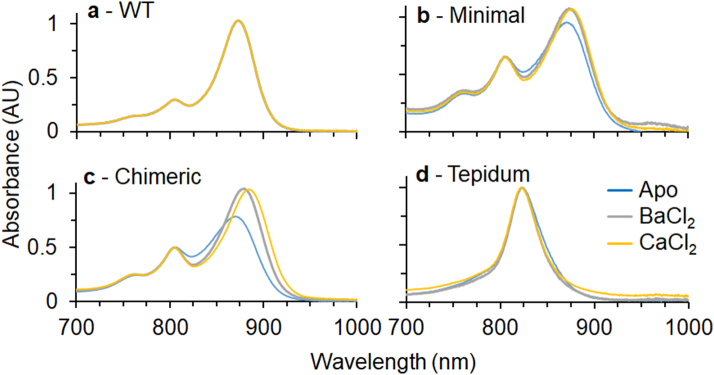
Absorbance spectra of purified WT RC-LH1 (a), Minimal RC-LH1 (b), Chimeric RC-LH1 (c) and Tepidum LH1 (d) complexes in the ion-free state (blue), or reconstituted with 5 mM BaCl_2_ (grey) or CaCl_2_ (yellow).

The *Tch. tepidum* RC-LH1 complex is able to bind strontium- and barium-ions, which initiate a smaller red-shift than calcium-ions. The binding sites for these ions are not identical to the calcium-ion site, which may explain why the level of red-shifting differs [60], [61]. To test for this ability in our engineered complexes, we removed calcium-ions on a chelating Sepharose column to ensure no chelating agents were present in the final sample. We then incubated our complex with excess BaCl_2_, SrCl_2_ or CaCl_2_. The resulting spectra are shown in Fig. 5. The data show that there were no observable differences in the WT complex. The Minimal and Chimeric constructs show a red-shift and increase in extinction upon calcium-ion binding. Reconstitution with BaCl_2_ leads to a similar increase in extinction and a smaller red-shift when compared to CaCl_2_. The spectra were identical to those for BaCl_2_ upon addition of SrCl_2_ (data not shown).

These properties are similar to that of the native *Tch. tepidum* RC-LH1, showing that our designed constructs have similar alternative ion-binding characteristics [43]. The spectra of isolated Tepidum LH1 are identical under all tested conditions, which suggests that the loss of characteristic LH1 absorbance, probably due to degradation of the complex upon ion-removal, is irreversible.

**Fig. 6:**
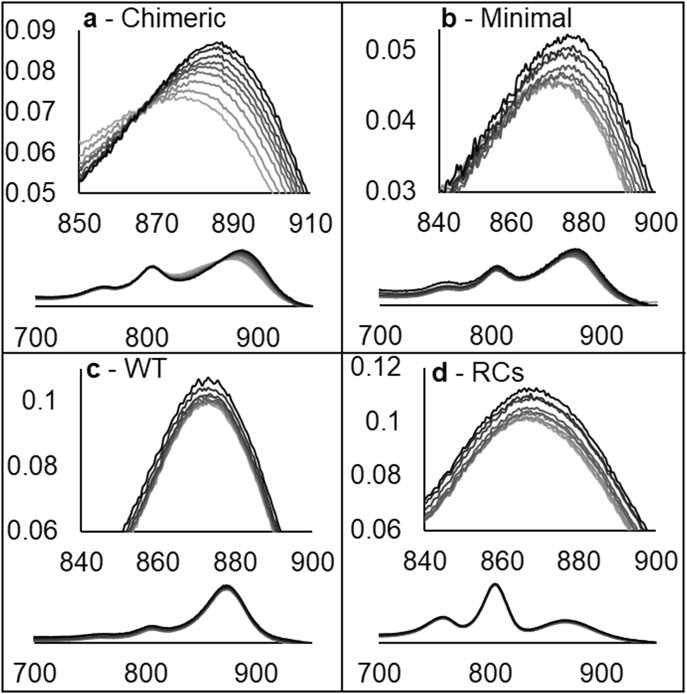
Spectra of purified Chimeric (a), Minimal (b), WT (c), and RCs (d) during titration of CaCl_2_ into solutions of pure ion-depleted complexes. Darker lines show increasing CaCl_2_ concentration from 0 to 48.13 μM. Main figures show the evolution of the LH1 Q_y_ peak with full-spectra shown beneath.

We measured the calcium-ion affinity of the Minimal and Chimeric complexes by titration of CaCl_2_ into the calcium-ion-depleted proteins and monitored the spectral evolution as the calcium concentration increased as shown in Fig. 6. In both of these constructs a red-shift and increase in extinction were observed. Control experiments with WT core complexes and purified RCs show only a small increase in absorbance, possibly a response of the RC primary donor to ionic strength, but no red-shift of the absorbance maximum. Plots of the A885 to A875 ratio from these spectra are shown in supplementary Fig. 4. As summarised in Table 3, fits to a single site binding model giving *K*_d_ values of 0.89 ± 0.32 μM and 1.48 ± 0.25 μM for purified Minimal and Chimeric complexes, respectively. Similar values were obtained for complexes in the native membrane. It should be noted that some error is associated with these measurements as the binding-site concentration is likely to be at-most one-fifth the *K*_d_ (estimated based upon the absorbance of the apo protein using the extinction coefficient of WT RC-LH1 multiplied by 16). Ideally the binding-site concentration should be less than one-tenth of the *K*_d_. Fitting of the pure Chimeric data to a model that accounts for this difference, assuming a binding-site concentration of 0.36 μM, yields a *K*_d_ value of 2 μM (data not shown). As both methods of fitting give similar values and the binding-site concentrations are only an estimate, the simple one-site model was used for all datasets. The estimated *K*_d_ values are remarkably similar to the 1.5 μM calcium-ion affinity published for the *Tch. tepidum* RC-LH1 complex [43] again showing that the biophysical properties of our engineered sites are similar.

**Table 3:** Dissociation constants measured for calcium-ion binding to complexes in native membranes and purified in β-DDM.

Sample	*K*_d_ membranes	*K*_d_ pure
WT	n/d^a^	n/d^a^
Minimal	0.89 ± 0.32 μM	0.83 ± 0.25 μM
Chimeric	1.48 ± 0.25 μM	1.10 ± 0.23 μM
Tepidum	n/d^b^	n/d^c^
RCs	n/d^d^	n/d^a^

a No response to calcium observed.

b No response to calcium removal.

c Protein unfolds during calcium-removal.

d Not measured.

**Fig. 7:**
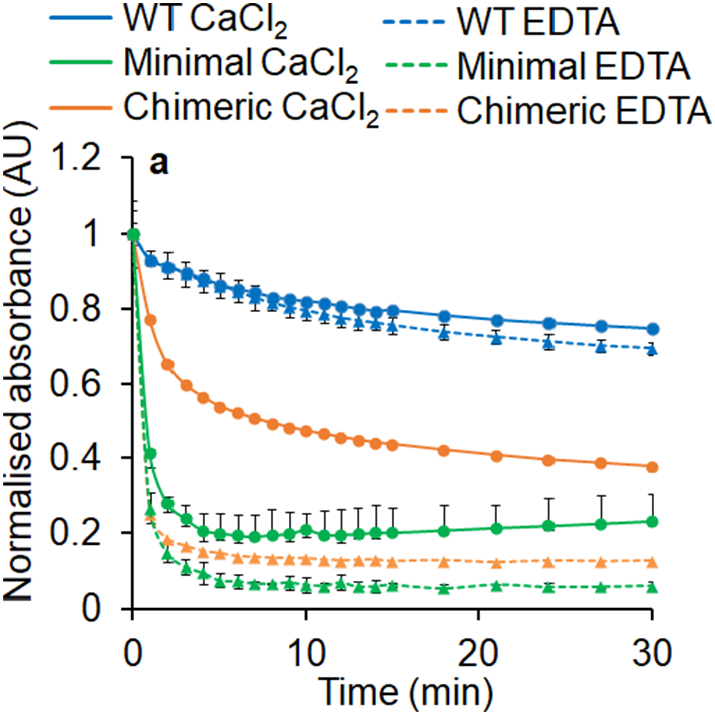
Thermal denaturation of WT (blue), Minimal (green) and Chimeric (orange) complexes in the presence of 5 mM CaCl_2_ (circles and solid lines) or 5 mM EDTA (triangles and dashed lines). Spectra are normalised to the initial absorbance of the Q_y_ maximum and were collected over 30 min at 55 °C. Each trace is an average of two or three data sets. Lines are included for clarity, error bars show standard error of the mean.

Finally, we examined the effect of calcium-ions on the thermal stability of our complexes. As shown in Fig. 7, incubating complexes at 55 °C and observing spectral evolution over 30 min allows monitoring of denaturation by loss of the LH1 Q_y_ band. The data show that the denaturation of Chimeric complexes is significantly faster when calcium-ions are removed with EDTA. The minimal construct shows very little, if any, enhancement in stability by calcium-ions, consistent with the insensitivity to calcium-ions during *in vitro* assembly. By contrast no significant difference is observed for WT complexes in excess CaCl_2_ or EDTA. These data show that, like in the *Tch. tepidum* RC-LH1 [42], calcium-ions significantly enhance the stability of these complexes. However, this level of enhancement seems correlated with the amount of *Tch. tepidum* sequence incorporated into the LH1 antenna.

Taken together, the ion-binding properties of our engineered complexes display characteristics similar to the *Tch. tepidum* RC-LH1 on which the designs were based. Despite the somewhat reduced degree of red-shifting, these data further demonstrate that ion-binding is fully functional in our designed Minimal and Chimeric complexes, and that the C-terminal domains from *Tch. tepidum* can be viewed as a module that can be imported into a foreign antenna complex, conferring calcium-ion binding and absorption shifting properties.

### 3.5 Ability of complexes to support photosynthetic growth

To examine the viability of our complexes to support photosynthetic growth, the designed constructs were used to replace the LH1 polypeptides of the WT strain which, unlike in our previous experiments, also assembles the peripheral LH2 antenna. Semi-aerobic starter cultures were prepared that had no clear differences in their growth as expected, as the photosynthetic complexes have no effect on cell viability under these conditions. When these cells were used to inoculate cultures under continuous illumination, marked differences in the growth rate became apparent. As shown in supplementary Fig. 5 all of the engineered strains supported slower growth than WT cells. This is likely to be a result of reduced expression of the LH1 antenna leading to a reduction of light-harvesting capacity. We observe that the Minimal construct reaches the limit of our measurement range (average OD680 of approximately 1.8) in around twice the time required by the WT. The Chimeric construct is further impaired requiring around five-times the period of WT. Finally the Tepidum construct shows the highest level of growth impediment requiring approximately six-times the period of WT to reach the end of the measurement limit. The Tepidum LH1 does not form a core complex, so the RCs are essentially disconnected from the antenna system and are only able to utilise a small proportion of the photons absorbed by the cell.

## 4 Conclusions

The data presented here show that we have been successful in engineering calcium-ion binding into the LH1 antenna of *Rba. sphaeroides* using carefully selected elements of the *Tch. tepidum* LH1 polypeptide sequences. In doing this we have avoided the issues caused by exchanging the LH1 antenna with that of *Tch. tepidum* that prevents proper assembly of the core complex. Although we have a reduced level of red-shifting in the Minimal and Chimeric constructs, the ability to form an intact core complex around the *Rba. sphaeroides* RC without replacing the native Crt, spheroidene, with the *Tch. tepidum* crt spirilloxanthin represents a good compromise. This work highlights the need to examine interactions between the core complex subunits and preserve these as much as possible during the design phase. These findings allow us to update our design strategies for the more effective creation of future modified antenna complexes.

These experiments also provide major insights into the unique red-shifted absorbance and thermal stability of the *Tch. tepidum* complex. Our data suggest that the exceptional thermal stability is dependent on interactions between LH1 and the RC whereas the red-shift is not. This is based on the fact that LH1 is very unstable when separated from the RC as apparently is the case in the Tepidum mutant whereas the red-shift is maintained when LH1 is not interacting with the RC or the RC is not present. Our data also show that without the incorporation of the RC, the *Tch. tepidum* LH1 is unable to undergo the required conformational changes to form the blue-shifted LH1 antenna complex. These findings highlight the requirement for a complete and stable core complex for a fully functional calcium-ion dependent spectral switch. Further investigation of our data shows that the full C-terminal regions of the *Tch. tepidum* α and β polypeptides are required for significant enhancements in thermal stability and of the red-shifting of absorbance in the calcium ion-bound state. We also find that features in the N-terminal domain, distant from the calcium-ion binding site of *Tch. tepidum* LH1 α and β, contribute to the large red-shift to 915 nm observed in its native RC-LH1 complex. The reconstitution experiments show the importance of spirilloxanthin binding to stabilise the formation of LH1 and cause a modest red-shift of the BChl Q_y_ band. Since the red-shift of the Q_y_ band is not as extensive in the reconstitution experiments as observed in *Tch. tepidum*, other factors enabling specific interactions in the N-terminal half of the α and β polypeptides or the presence of specific lipid components may be important. Such factors will be a focus of our future research to fully understand the interactions underlying the red-shift and exceptional stability of the LH1-RC core complex in *Tch. tepidum*. Applications for the utilisation of our engineered complexes for both *in vivo* studies and in biohybrid systems are also currently being explored.

## Author contributions

DJKS, PAL and CNH designed the experiments. DJKS, ECM, CV, PSPL and PAL collected data, and all authors contributed to preparation of the manuscript.

## Transparency document

Transparency document. Image 2

## Appendix A Supplementary data

Supplementary material Image 3
