# Supplementary material for: Engineering of a calcium-ion binding site into the RC-LH1-PufX complex of Rhodobacter sphaeroides to enable ion-dependent spectral red-shifting
Source: Biochim Biophys Acta. 2017 Nov;1858(11):927–38. doi: 10.1016/j.bbabio.2017.08.009 (PMC5604489; doi:10.1016/j.bbabio.2017.08.009)
Supplement: Supplementary file 2 — Supplementary material [file mmc2.docx]

**Supplementary data**

**Supplementary Table 1.** DNA sequences of GBlocks for the production of pK18MOBSAC vectors containing designed sequences

| Construct | GBlock Sequence |
| --- | --- |
| Minimal | CGCGCCTGACCGCAGGTCAGGTTGCGACACGCCATTCGTCGTCTCCCCAAGGGGCGGCGGATTAATCGGGAGGGCATGGTGCCTTACCGTAACCCACGCCACCAGCATGTGGCGTCAGTCTTACGATCCGGAGGATAGCATGGCTGATAAATCCGACCTGGGCTACACAGGTCTTACGGACGAGCAGGCGCAGGAATTGCACTCCGTCTACATGAGCGGTCTTTGGCTGTTCAGCGCGGTGGCGATCGTCGCTCACCTGTTGGCCTGGCTGTATCGTCCCTGGCTGTGAGGAGAACAAGATCATGAGCAAGTTCTACAAAATTTGGATGATCTTCGATCCCCGTCGCGTGTTCGTGGCTCAGGGCGTGTTCCTGTTCCTCCTCGCGGTGATGATCCACATGATCGTGCTGAGCACCGACCTCAACTGGCTGGACGACAACGCCGCCAAGTACAACCGCGTCGCCGTCGCCGAGTAATCGGGCCGGCCCTCCGTC |
| Chimeric | CGCGCCTGACCGCAGGTCAGGTTGCGACACGCCATTCGTCGTCTCCCCAAGGGGCGGCGGATTAATCGGGAGGGCATGGTGCCTTACCGTAACCCACGCCACCAGCATGTGGCGTCAGTCTTACGATCCGGAGGATAGCATGGCTGATAAATCCGACCTGGGCTACACAGGTCTTACGGACGAGCAGGCGCAGGAATTGCACTCCGTCTACATGAGCGGTCTTTGGCTGTTCAGCGCGGTGGCGATCGTCGCTCACCTGTTGGCCTGGCTGTATCGTCCCTGGCTGTGAGGAGAACAAGATCATGAGCAAGTTCTACAAAATTTGGATGATCTTCGATCCCCGTCGCGTGTTCGTGGCTCAGGGCGTGTTCCTGTTCCTCCTCGCGGTGATGATCCACATGATCGTGCTCAGCACCGATCTGAACTGGCTGGATGACAATATCCCCGTGAGCTATCAGGCGCTGGGCAAGAAATAATCGGGCCGGCCCTCCGTC |
| Tepidum | CGCGCCTGACCGCAGGTCAGGTTGCGACACGCCATTCGTCGTCTCCCCAAGGGGCGGCGGATTAATCGGGAGGGCATGGTGCCTTACCGTAACCCACGCCACCAGCATGTGGCGTCAGTCTTACGATCCGGAGGATAGCATGGCTGAACAGAAGAGCTTGACCGGCTTGACCGACGACGAGGCGAAGGAGTTCCACGCGATCTTCATGCAGAGCATGTATGCGTGGTTTGGCCTGGTCGTGATCGCCCACCTGTTGGCCTGGCTGTATCGTCCCTGGCTGTGAGGAGAACAAGATCATGTTCACGATGAATGCAAATCTTTATAAGATCTGGCTCATCCTCGATCCGCGGCGGGTGCTGGTGTCGATCGTTGCGTTCCAGATCGTCCTGGGCCTGCTCATCCATATGATCGTGCTCAGCACCGATCTGAACTGGCTGGATGACAATATCCCCGTGAGCTATCAGGCGCTGGGCAAGAAATAATCGGGCCGGCCCTCCGTC |


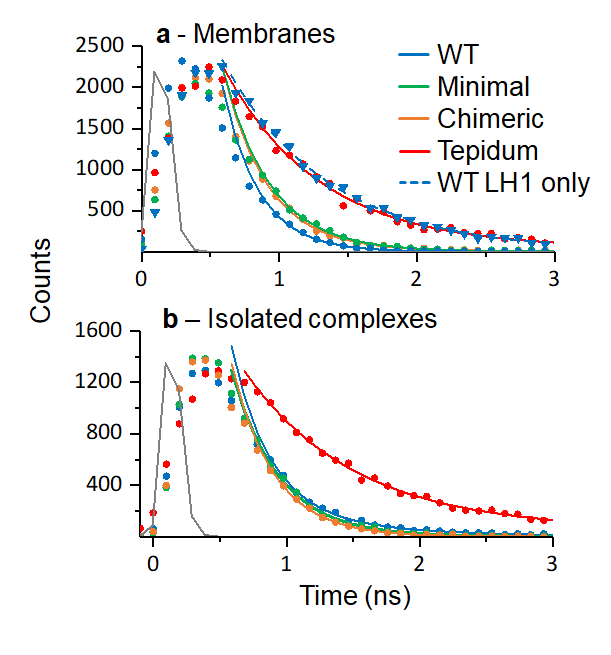


**Supplementary figure 1**. Fluorescence lifetime traces for membranes (a) and purified complexes (b). Points show raw data and solid lines show fits for WT RC-LH1 (blue, solid line and circles), Minimal (green), Chimeric (Orange), Tepidum (Red) and LH1-only membranes (blue, dashed line and triangles). All data were fit to a single exponential with the exception of WT isolated complexes, which was fit to a two exponent model. The grey curve shows the instrument response function.


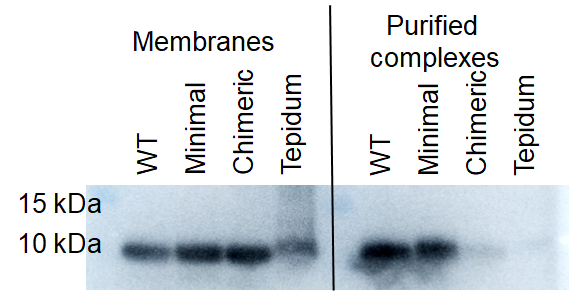


**Supplementary figure 2**. Immunodetection of pufX in LH2 deficient membranes containing WT, Minimal, Chimeric and Tepidum LH1, and in purified protein complexes.


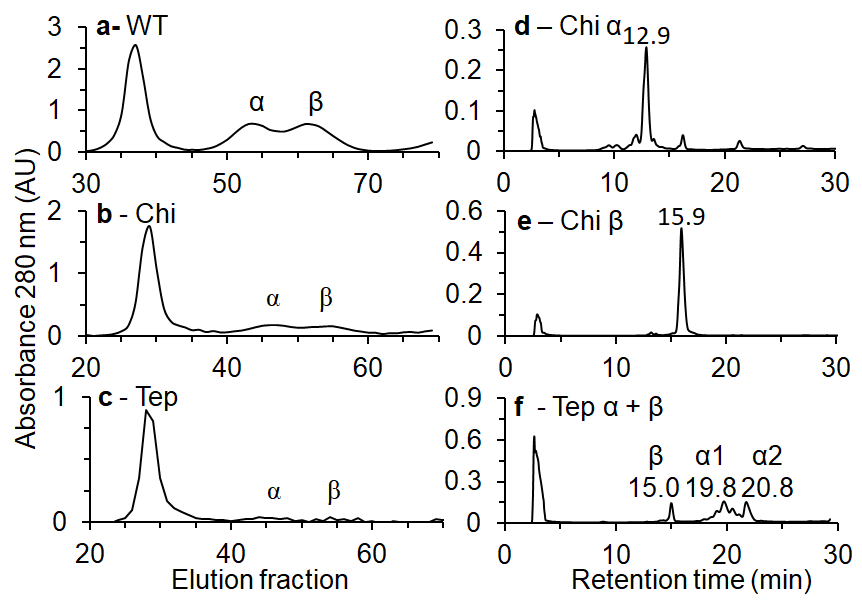


**Supplementary figure 3.** a-c, polypeptide and pigment separation on a Sephadex LH60 column for a: WT (void volume at 37 min and pigments elution after 75 min), b: Chimeric (void volume at 29 min and pigments elution after 70 min) and c: Tepidum (void volume at 28 min and pigments elution after 70 min). The positions of the α and β polypeptide peaks are labelled on each trace. d-f, HPLC purification for d: Chimeric the α polypeptide, e: Chimeric β and f: Tepidum α and β. Peaks are labelled with their respective retention times.


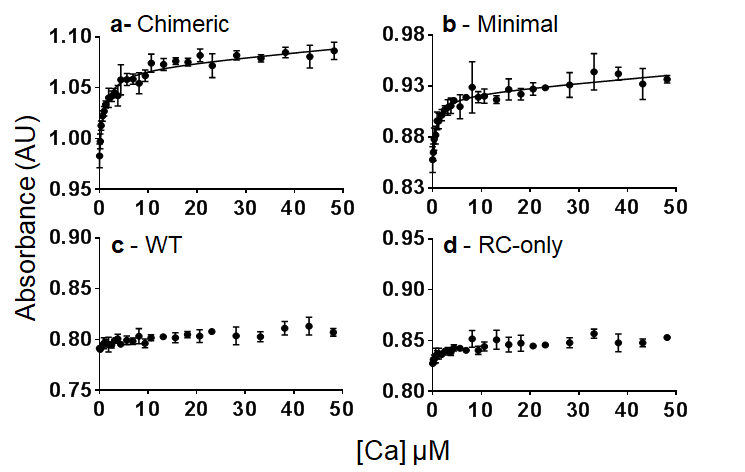


**Supplementary figure 4**. Plots of the A885/A875 ratio against CaCl_2_ concentration for the purified Chimeric (a), Minimal (b), WT (c) RC-LH1 constructs, and pure RCs (d) shown in figure 5. Error bars show standard deviations for each data point and solid lines show fits to a one-site binding model for the Chimeric and Minimal datasets.


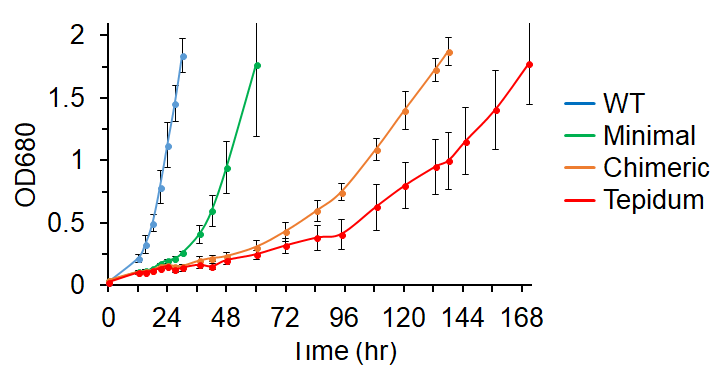


**Supplementary figure 5**. Growth curves of WT, Minimal, Chimeric and Tepidum expressing strains of *Rba. sphaeroides* with native LH2 expression following OD680 during photosynthetic growth. Error bars show standard deviation of three cultures for each strain. Lines between points have been added for clarity.
